# Azacitidine improves clinical outcomes in older patients with acute myeloid leukaemia with myelodysplasia-related changes compared with conventional care regimens

**DOI:** 10.1186/s12885-017-3803-6

**Published:** 2017-12-14

**Authors:** John F. Seymour, Hartmut Döhner, Aleksandra Butrym, Agnieszka Wierzbowska, Dominik Selleslag, Jun Ho Jang, Rajat Kumar, James Cavenagh, Andre C. Schuh, Anna Candoni, Christian Récher, Irwindeep Sandhu, Teresa Bernal del Castillo, Haifa Kathrin Al-Ali, Jose Falantes, Richard M. Stone, Mark D. Minden, Jerry Weaver, Steve Songer, C. L. Beach, Hervé Dombret

**Affiliations:** 1 Department of Haematology, Peter MacCallum Cancer Centre, Locked Bag 1, A’Beckett St, East Melbourne, VIC 8006 Australia; 2 University of Melbourne, Parkville, Australia; 3 Universitätsklinikum Ulm, Ulm, Germany; 4 Wroclaw Medical University, Wroclaw, Poland; 5 Medical University of Lodz, Lodz, Poland; 6 Algemeen Ziekenhuis Sint-Jan, Brugge, Belgium; 7 Samsung Medical Center, Sungkyunkwan University School of Medicine, Seoul, South Korea; 8 Cancer Care Manitoba, Winnipeg, Canada; 9 Barts Health National Health Service Trust, London, UK; 10 Princess Margaret Cancer Centre, Toronto, Canada; 11 Azienda Sanitaria Universitaria Integrata di Udine, Udine, Italy; 12 Centre Hospitalier Universitaire de Toulouse, Toulouse, France; 13 University of Alberta Hospital, Edmonton, Canada; 14 Hospital Central de Asturias, Oviedo, Spain; 15 University Hospital of Halle, Halle, Germany; 16 Hospital Universitario Virgen del Rocio/Instituto de BioMedicinia de Sevilla, Sevilla, Spain; 17 Dana-Farber Cancer Institute, Boston, MA USA; 18 Celgene Corporation, Summit, NJ USA; 19 Hôpital Saint Louis, Institut Universitaire d’Hématologie, University Paris Diderot, Paris, France

**Keywords:** Azacitidine, Low-dose cytarabine, Acute myeloid leukaemia, AML, Myelodysplasia-related changes, AML-MRC, Induction chemotherapy, Response, Survival

## Abstract

**Background:**

Compared with World Health Organization-defined acute myeloid leukaemia (AML) not otherwise specified, patients with AML with myelodysplasia-related changes (AML-MRC) are generally older and more likely to have poor-risk cytogenetics, leading to poor response and prognosis. More than one-half of all older (≥65 years) patients in the phase 3 AZA-AML-001 trial had newly diagnosed AML-MRC.

**Methods:**

We compared clinical outcomes for patients with AML-MRC treated with azacitidine or conventional care regimens (CCR; induction chemotherapy, low-dose cytarabine, or supportive care only) overall and within patient subgroups defined by cytogenetic risk (intermediate or poor) and age (65–74 years or ≥75 years). The same analyses were used to compare azacitidine with low-dose cytarabine in patients who had been preselected to low-dose cytarabine before they were randomized to receive azacitidine or CCR (ie, low-dose cytarabine).

**Results:**

Median overall survival was significantly prolonged with azacitidine (*n* = 129) versus CCR (*n* = 133): 8.9 versus 4.9 months (hazard ratio 0.74, [95%CI 0.57, 0.97]). Among patients with intermediate-risk cytogenetics, median overall survival with azacitidine was 16.4 months, and with CCR was 8.9 months (hazard ratio 0.73 [95%CI 0.48, 1.10]). Median overall survival was significantly improved for patients ages 65–74 years treated with azacitidine compared with those who received CCR (14.2 versus 7.3 months, respectively; hazard ratio 0.64 [95%CI 0.42, 0.97]). Within the subgroup of patients preselected to low-dose cytarabine before randomization, median overall survival with azacitidine was 9.5 months versus 4.6 months with low-dose cytarabine (hazard ratio 0.77 [95%CI 0.55, 1.09]). Within the low-dose cytarabine preselection group, patients with intermediate-risk cytogenetics who received azacitidine had a median overall survival of 14.1 months versus 6.4 months with low-dose cytarabine, and patients aged 65–74 years had median survival of 14.9 months versus 5.2 months, respectively. Overall response rates were similar with azacitidine and CCR (24.8% and 17.3%, respectively), but higher with azacitidine versus low-dose cytarabine (27.2% and 13.9%). Adverse events were generally comparable between the treatment arms.

**Conclusions:**

Azacitidine may be the preferred treatment for patients with AML-MRC who are not candidates for intensive chemotherapy, particularly patients ages 65–74 years and those with intermediate-risk cytogenetics.

**Trial registration:**

This study was registered at clinicalTrials.gov on February 16, 2010 (NCT01074047).

## Background

Acute myeloid leukaemia (AML) is a heterogeneous disorder with multifactorial pathogenic mechanisms [1]. AML pathogenesis is characterised by recurrent chromosomal translocations and specific somatic mutations that define biologically distinct disease subtypes. The World Health Organization (WHO) classification of myeloid neoplasms includes four distinct types of AML [2]: AML with recurrent genetic abnormalities; therapy-related AML (tAML); AML with myelodysplasia-related changes (AML-MRC); and AML not otherwise specified (AML-NOS). Among WHO-defined AML classifications, the most common are AML-MRC and AML-NOS [3]. AML-MRC is defined by the presence of multilineage dysplasia, prior history of myelodysplastic syndromes (MDS) (secondary AML [sAML]), or MDS-related cytogenetic abnormalities [4]. Compared with AML-NOS, patients with AML-MRC are generally older and more likely to have poor-risk cytogenetics, leading to poor response and prognosis [5]. Moreover, older patients with sAML tend to have disease that is more chemoresistant than do patients with AML-NOS [6]. While choice of AML treatment can depend on age, cytogenetic risk, performance status, and other factors, no particular therapy has yet been found to provide specific benefits in AML-MRC.

In the United States, azacitidine is recommended first-line therapy for patients with higher-risk MDS [7], and is indicated for all FAB subtypes of MDS and for treatment of low-blast-count AML (20–30% bone marrow [BM] blasts) [8]. Azacitidine is also indicated in the European Union for higher-risk MDS and for treatment of adult patients with AML with any BM blast count who are unable to undergo allogeneic stem cell transplantation [9]. MDS and AML may reflect a continuum of myeloid disease, particularly in the older patient population with low white blood cell (WBC) counts. The international, randomised, phase 3 AZA-AML-001 (AZA-AML) study in older patients with newly diagnosed AML with >30% BM blasts showed overall survival (OS) was 10.4 months in azacitidine-treated patients compared with 6.5 months for patients who received conventional care regimens (CCR; *P* = 0.101) [10]. Consistent with azacitidine efficacy in higher-risk MDS, a prospective univariate analysis of 158 patients (32% of all patients in the AZA-AML study) with locally diagnosed AML-MRC showed that azacitidine was associated with significantly improved OS compared with CCR (*P* = 0.036), and the difference approached statistical significance in multivariate analysis (*P* = 0.097) [10]. Subsequently, central review of patients’ BM samples identified a much larger proportion of patients who met WHO criteria for AML-MRC, suggesting the possibility of substantial under-diagnosis of AML-MRC in routine clinical practise. To help clarify whether patients with AML with myelodysplasia-related features respond preferentially to azacitidine, we evaluated efficacy and safety outcomes for this larger group of patients with centrally adjudicated AML-MRC in the AZA-AML trial.

## Methods

The AZA-AML study was approved by the relevant institutional review boards or independent ethics committees and was conducted according to the Declaration of Helsinki. All patients provided written informed consent before study participation.

### Patients

Inclusion and exclusion criteria and study design are reported in detail elsewhere [10]. Briefly, patients aged ≥65 years with newly diagnosed de novo AML or sAML with >30% BM blasts, Eastern Cooperative Oncology Group performance status (ECOG PS) scores 0–2, intermediate- or poor-risk cytogenetics per National Comprehensive Cancer Network (NCCN) 2009 criteria, and WBC counts ≤15 × 10^9^/L were eligible. Before randomisation, patients were preselected to 1 of 3 protocol-specified CCR: intensive chemotherapy (IC; standard 7 + 3 regimen with one induction course and up to two additional courses of consolidation), low-dose cytarabine (LDAC; 20 mg BID for 10 days per 28-day treatment cycle), or best supportive care (BSC) only. Patients were then randomised 1:1 to azacitidine (75 mg/m^2^/day for 7 days per 28-day cycle) or to CCR. Those randomised to CCR received their preselected treatment. All participants could receive BSC as needed.

Diagnosis and disease classification were recorded before study entry for each patient by the treating physician. Subsequently, assignment of AML-MRC according to WHO criteria was performed by the sponsor. Patients with centrally adjudicated AML-MRC must have met at least one of the following criteria: (1) dysplasia in ≥50% of cells in at least two of the three myeloid lineages, (2) sAML with antecedent history of MDS or myeloproliferative neoplasms (MPN), or (3) MDS-related cytogenetic abnormalities [4]. Dysplasia and cytogenetic assessments of baseline BM aspirate samples were performed centrally by an independent expert pathologist (John M. Bennett, MD) and cytogeneticist (Anne Hagemeijer, MD), blinded to local pathology and cytogenetic reports and treatment assignments. Proportions of dysplastic cells in the erythroid, granulocytic, and megakaryocytic lineages were determined by independent haemato-pathologist review of BM aspirates. History of MDS was based on local site reporting. Patients who met criteria for AML-MRC but who had received prior systemic anti-cancer therapy or prior radiation treatment were considered to have tAML per WHO definition and were excluded from analyses.

### Efficacy endpoints

Analyses included only those patients in AZA-AML with a centrally confirmed AML-MRC diagnosis. The primary analysis compared the OS for azacitidine- and CCR-treated patients. Additionally, a sensitivity analysis of OS, similar to one that was predefined for the overall AZA-AML study [10], was performed, in which patients who had switched to alternate therapy at some point from their randomised treatment were censored at the time they did so.

Secondary analyses included Kaplan-Meier estimates of 1-year survival and overall response rate (i.e., complete remission [CR] plus CR with incomplete haematologic recovery [CRi]) as defined by International Working Group (IWG) response criteria for AML [11]. Stable disease was defined as the absence of an IWG-defined hematologic response with no evidence of disease progression, sustained for a period of ≥8 weeks. Disease progression was defined as 1) > 50% increase in BM blast count percentage from baseline that persisted for ≥2 BM assessments separated by ≥1 month, unless the baseline count was >70%, in which case, >70% blasts persisting for 2 BM assessments separated by ≥1 month; or 2) doubling of the baseline peripheral blood blast count that persisted for ≥7 days with final peripheral blood blast count >10 × 10^9^/L. OS outcomes with azacitidine and CCR were also evaluated within patient subgroups defined by cytogenetic risk (intermediate or poor) and by age (65–74 years or ≥75 years).

Of all 488 patients enrolled in AZA-AML, 312 (64%) were preselected to LDAC before randomisation to treatment; IC and BSC-only were each preselected for 18% of patients [10]. Because LDAC and azacitidine are both lower-intensity active therapies, OS and haematologic response analyses were also performed within the subgroup of patients with AML-MRC preselected to LDAC before randomisation, who then received azacitidine or LDAC on-study. Additionally, as IC was the other active CCR treatment option, OS and response were compared for patients preselected to IC who later received azacitidine or IC.

Exploratory analyses compared OS with azacitidine or CCR in patients who met only a single AML-MRC criterion (e.g., multilineage dysplasia but no history of prior MDS or MDS-related cytogenetics).

### Safety

The safety population included all patients who received at least 1 dose of study drug (or at randomisation for the BSC-only group) and had at least 1 safety assessment thereafter. Treatment-emergent adverse events (TEAEs) were defined as new or worsening AEs between the time of first dose (or at randomisation for BSC-only patients) until 28 days after the last dose of azacitidine or LDAC, 70 days after the last dose of IC, or the day of discontinuation and/or study closure for patients receiving BSC only. TEAEs were coded by Medical Dictionary for Regulatory Activities (MedDRA), and graded for severity using the National Cancer Institute Common Toxicity Criteria for Adverse Events (NCI-CTCAE) version 4.0.

### Statistical methods

Demographic and disease characteristics at baseline are reported descriptively. Median OS and 1-year survival rates were determined based on Kaplan-Meier product limit estimates. Hazard ratios (HR) and 95% CI for OS are estimated from unstratified Cox proportional hazards models. As these OS analyses are post hoc in nature, multiplicity of testing and power considerations did not allow for a proper interpretation of *P* values; therefore, results are presented as point estimates with corresponding 95% confidence intervals (CI). To assess heterogeneity, 95% CI for OS within individual CCR arms (IC, LDAC, and BSC) were constructed. Overall response rate was compared between azacitidine and CCR using summary statistics.

## Results

### Baseline demographic and disease characteristics

In all, 262 patients (54% of all patients in AZA-AML) fulfilled WHO criteria for AML-MRC upon central review; 129 were treated with azacitidine and 133 received CCR (IC *n* = 24, LDAC *n* = 79, BSC only *n* = 30). This AML-MRC subgroup represents 53% of all azacitidine-treated patients and 54% of all CCR patients in the AZA-AML study. Interestingly, of all patients in the CCR arm who received BSC only in the AZA-AML study, 67% (30/45) had AML-MRC, suggesting AML-MRC patients were frequently considered to be at higher-risk by their managing physicians during treatment preselection.

**Fig. 1:**
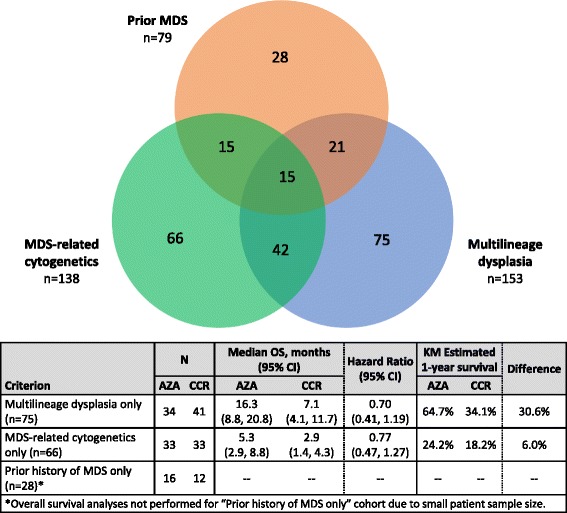
Patient distribution according to WHO AML-MRC criteria

In both treatment arms, median ages were greater than 75 years, and approximately one-half of all patients had poor-risk cytogenetics (Table 1). Ninety-three patients (35%) met two or more AML-MRC criteria, including 15 patients (6%) who met all three criteria (Fig. 1). Of 87 patients identified locally as having a prior diagnosis of MDS before study entry, 8 were not classified as AML-MRC in the central review because they met at least one WHO-defined exclusion criterion (e.g., prior systemic anti-cancer treatment). In all, among 262 patients with centrally defined AML-MRC, 79 (30%) had had prior MDS, 138 (53%) had MDS-related cytogenetic abnormalities, and 153 (58%) had morphologic multilineage dysplasia. Notably, of the 153 patients determined to have multilineage dysplasia based on central review of BM aspirates, 89 patients were originally reported to have had no or only one dysplastic lineage according to local assessment, and 66 of these 89 patients (74%) were not classified as having AML-MRC at local diagnosis.

**Table 1:** Baseline demographic and disease characteristics

	All patients with AML-MRC (*N* = 262)		LDAC-preselected patients with AML-MRC (*n* = 160)	
	Azacitidine (*n* = 129)	CCR (*n* = 133)	Azacitidine (*n* = 81)	LDAC (*n* = 79)
Age (years), median (min, max)	76 (64, 90)	75 (65, 87)	76 (64, 90)	75 (65, 87)
Age ≥ 75 years, *n* (%)	77 (60)	69 (52)	55 (68)	41 (52)
Male gender, *n* %	81 (63)	78 (59)	42 (52)	43 (54)
Prior history of MDS*, *n* (%)	44 (34)	35 (26)	32 (40)	20 (25)
ECOG PS, *n* (%)				
0–1	94 (73)	104 (78)	56 (69)	64 (81)
2	35 (27)	29 (22)	25 (31)	15 (19)
Cytogenetic risk, *n* (%)				
Intermediate	63 (49)	61 (46)	47 (58)	33 (42)
Poor	66 (51)	72 (54)	34 (42)	46 (58)
No. of dysplastic lineages, *n* (%)				
0–1	57 (44)	52 (39)	36 (44)	35 (44)
2–3	72 (56)	81 (61)	45 (56)	44 (56)
% BM blasts, median (min, max)	65.0 (27, 99)	70.0 (26, 100)	66.0 (27, 99)	69.0 (31, 100)
Haematology, median (min, max)				
ANC (10^9^/L)	0.4 (0.0, 11.6)	0.3 (0.0, 8.7)	0.4 (0.0, 11.6)	0.3 (0.0, 8.7)
Platelets (10^9^/L)	56 (3, 585)	55 (6, 244)	58 (7, 585)	55 (6, 244)
WBC (10^9^/L)	3.2 (0.6, 26.5)	2.4 (0.4, 22.6)	2.6 (0.6, 26.5)	2.3 (0.4, 13.5)
Hgb (g/dL)	9.5 (5.0, 13.4)	9.3 (5.0, 14.4)	9.4 (5.0, 11.8)	9.4 (5.6, 14.4)

*Based on local site reporting

AML-MRC, AML with myelodysplasia-related changes; ANC, absolute neutrophil count; BM, bone marrow; CCR, conventional care regimens; ECOG PS, Eastern Cooperative Oncology Group performance status; Hgb, haemoglobin; LDAC, low-dose cytarabine; MDS, myelodysplastic syndromes; WBC, white blood cell count

The majority of the AML-MRC population (160/262; 61%) had been preselected to LDAC before study randomisation. Baseline characteristics of LDAC-preselected patients who ultimately received azacitidine (*n* = 81) or LDAC (*n* = 79) were generally comparable (Table 1), although proportionately more azacitidine-treated patients had an ECOG PS score of 2 (31% vs 19% of patients treated with LDAC) and proportionately more LDAC-treated patients had poor-risk cytogenetics (58% vs 42% of patients treated with azacitidine). Azacitidine-treated patients also tended to be older; 68% of patients in this preselection group were aged ≥75 years, compared with 52% of LDAC-treated patients.

Patients in the azacitidine arm received a median of five treatment cycles (range 1–27), and those in the CCR arm received a median of two IC treatment cycles (1–3), two LDAC treatment cycles (1–22), or three 28-day cycles of BSC-only (1–9). For patients preselected to receive LDAC but treated with azacitidine, the median number of azacitidine treatment cycles was six (range 1–25).

### Survival: All AML-MRC patients

**Fig. 2:**
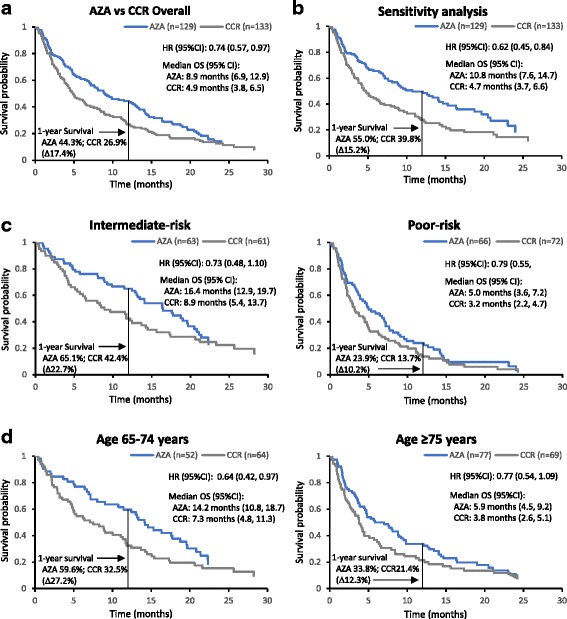
Overall survival among patients with AML-MRC. **a** Overall survival for all patients with AML-MRC treated with azacitidine or CCR. **b** Sensitivity analysis, in which patients who switched to alternate therapy were censored at the time they did so. **c** Overall survival by NCCN cytogenetic risk. **d** Overall survival by age group

Median OS among all patients with AML-MRC was significantly prolonged in the azacitidine arm compared with the CCR arm (8.9 vs 4.9 months, respectively; HR 0.74, 95%CI 0.57, 0.97) (Table 2; Fig. 2a), and the estimated 1-year survival rate was 17.4% greater in the azacitidine arm (44.3% vs 27.2% with CCR). In the survival sensitivity analysis, in which patients were censored at the time they received alternate therapy, median OS increased in both groups, to 11.4 months for azacitidine-treated patients and to 5.4 months for patients who received CCR (HR 0.72, 95%CI 0.52, 1.0); 1-year survival estimates were 49.4% *vs.* 31.0%, respectively (Fig. 2b). There was no evidence of significant heterogeneity of treatment effect among the 3 CCR treatments; median OS in the IC, LDAC, and BSC-only groups were 8.9 months (95%CI 3.2, 15.1), 4.6 months (3.3, 6.4), and 3.8 months (2.0, 8.0), respectively. Within the subgroup of patients preselected to IC, median OS in IC-preselected patients who received azacitidine (*n* = 22) was slightly improved at 11.6 months compared with 8.9 months among IC-treated patients (*n* = 24), but the difference was not statistically significant (HR 0.83, 95%CI 0.42, 1.62).

**Table 2:** Survival outcomes among all patients with AML-MRC (N = 262) and among those preselected to LDAC (n = 160)

All patients with AML-MRC (N = 262)									
	*N*		Median OS, months (95%CI)		Difference, months	Hazard ratio (95% CI)	K-M Estimated 1-Year survival		Difference
	AZA	CCR	AZA	CCR			AZA	CCR	
Overall	129	133	8.9 (6.9, 12.9)	4.9 (3.8, 6.5)	4.0	**0.74 (0.57, 0.97)**	44.3%	26.9%	17.4%
Cytogenetic risk									
Intermediate	63	61	16.4 (12.9, 19.7)	8.9 (5.4, 13.7)	7.5	0.73 (0.48, 1.10)	65.1%	42.4%	22.7%
Poor	66	72	5.0 (3.6, 7.2)	3.2 (2.2, 4.7)	1.8	0.79 (0.55, 1.11)	23.9%	13.7%	10.2%
Age									
65–74 years	52	64	14.2 (10.8, 18.7)	7.3 (4.8, 11.3)	6.9	**0.64 (0.42, 0.97)**	59.6%	32.5%	27.2%
≥75 years	77	69	5.9 (4.5, 9.2)	3.8 (2.6, 5.1)	2.1	0.77 (0.54, 1.09)	33.8%	21.4%	12.3%
LDAC-preselected patients with AML-MRC (n = 160)									
	*N*		Median OS, months (95%CI)		Difference, months	Hazard ratio (95% CI)	K-M Estimated 1-Year survival		Difference
	AZA	LDAC	AZA	LDAC			AZA	LDAC	
Overall	81	79	9.5 (5.9, 14.1)	4.6 (3.3, 6.4)	4.9	0.77 (0.55, 1.09)	45.3%	23.5%	21.8%
Cytogenetic risk									
Intermediate	47	33	14.1 (8.9, 17.6)	6.4 (3.8, 14.2)	7.7	0.90 (0.54, 1.50)	57.4%	34.4%	23.0%
Poor	34	46	5.6 (2.2, 9.5)	3.7 (2.2, 5.1)	1.9	0.83 (0.52, 1.33)	28.1%	15.8%	12.3%
Age									
65–74 years	26	38	14.9 (9.0, 19.6)	5.2 (3.5, 10.0)	9.7	0.68 (0.39, 1.18)	61.5%	26.3%	35.2%
≥75 years	55	41	8.8 (4.5, 12.9)	4.0 (2.8, 6.4)	4.8	0.78 (0.50, 1.22)	37.5%	20.8%	16.7%

AML-MRC, AML with myelodysplasia-related changes; AZA, azacitidine; CCR, conventional care regimens; CI, confidence interval; K-M, Kaplan-Meier

Hazard ratios in bold indicate statistical significance

Though the subgroup of patients with prior MDS as their sole AML-MRC criterion was too small for meaningful comparisons (*n* = 28), patients who only met the AML-MRC criterion of morphologic multilineage dysplasia (*n* = 75: azacitidine *n* = 34; CCR *n* = 41) had better OS outcomes than the group of all AML-MRC patients: median OS with azacitidine was 16.3 months vs 7.1 months with CCR (HR 0.70, 95%CI 0.41, 1.2), and estimated 1-year survival rates were 64.7% vs 34.1%, respectively. In contrast, patients whose only AML-MRC feature was MDS-related cytogenetics (AZA *n* = 33, CCR n = 33) fared much worse than overall: median OS with azacitidine vs CCR was 5.3 vs 2.9 months, respectively (HR 0.77, 95%CI 0.47, 1.3), and 1-year survival was 24.2% vs 18.2%.

Overall, 124 patients (47%) had intermediate-risk cytogenetics and 138 patients (53%) had poor-risk cytogenetics. For patients with intermediate-risk cytogenetics treated with azacitidine, median OS was 16.4 months compared with 8.9 months among patients treated with CCR (HR 0.73, 95%CI 0.48, 1.1) (Fig. 2c), with estimated 1-year survival rates of 65.1% and 42.4%, respectively (Table 2). As expected, poor-risk cytogenetics was associated with substantially worse prognosis; median OS with azacitidine was 5.0 months compared with 3.2 months with CCR (HR 0.79, 95%CI 0.55, 1.1) and estimated 1-year survival rates were 23.9% and 13.7%, respectively (Table 2 and Fig. 2c).

Overall, 116 patients were aged 65–74 years and 146 patients were aged ≥75 years (azacitidine age range 75–90 years; CCR age range 75–87 years). In the younger cohort, median OS among patients treated with azacitidine (*n* = 52) was significantly prolonged compared with that for CCR-treated patients (*n* = 64: LDAC *n* = 38, IC *n* = 18; BSC only *n* = 8): 14.2 vs 7.3 months, respectively (HR 0.64, 95%CI 0.42, 0.97) (Fig. 2d), with estimated 1-year survival rates of 59.6% for patients treated with azacitidine and 32.5% for those who received CCR (Table 2). Among those aged ≥75 years, median OS was 5.9 months with azacitidine (*n* = 77) and 3.8 months with CCR (*n* = 69: LDAC *n* = 41; IC n = 6; BSC only *n* = 22) (HR 0.77, 95%CI 0.54, 1.09) (Table 2).

### Survival: LDAC preselection subgroup

**Fig. 3:**
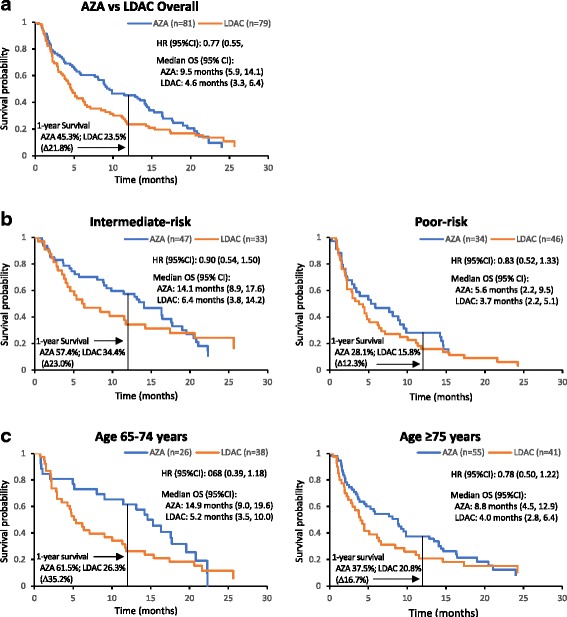
Overall survival among patients with AML-MRC preselected to LDAC. **a** Overall survival for patients with AML-MRC treated with azacitidine or LDAC; **b** Overall survival by cytogenetic risk; **c** Overall survival by age group

Median OS among LDAC-preselected patients treated with azacitidine was double that for LDAC-treated patients (9.5 vs 4.6 months, respectively; HR 0.77, 95%CI 0.55, 1.09) (Fig. 3a), and estimated 1-year survival rates were 45.3% vs 23.5%, respectively (Table 2).

Equal numbers of patients preselected to receive LDAC had NCCN-defined intermediate-risk cytogenetics (*n* = 80; azacitidine *n* = 47, LDAC *n* = 33) and poor-risk cytogenetics (n = 80; azacitidine *n* = 34, LDAC *n* = 46). Among patients with intermediate-risk cytogenetics, azacitidine treatment was associated with a 7.7-month improvement in median OS compared with LDAC: 14.1 vs 6.4 months, respectively (HR 0.90, 95%CI 0.54, 1.50) (Fig. 3b and Table 2). For patients with poor-risk cytogenetics, median OS with azacitidine *vs.* LDAC was 5.6 *vs.* 3.7 months, respectively (HR 0.83, 95%CI 0.52, 1.33) (Table 2).

Patients aged 65–74 years treated with azacitidine (*n* = 26) fared better than those treated with LDAC (n = 38), with a 9.7-month improvement in median OS: 14.9 vs 5.2 months, respectively (HR 0.68, 95%CI 0.39, 1.18) (Fig. 3c and Table 2). Among patients aged ≥75 years (*n* = 55), median OS was more than doubled in azacitidine-treated patients compared with LDAC-treated patients (*n* = 41) at 8.8 *vs*. 4.0 months, respectively (HR 0.78, 95%CI 0.50, 1.22) (Table 2).

### Response

Overall response rates (i.e., proportion of patients with CR or CRi) were 24.8% and 17.3% with azacitidine and CCR, respectively. Despite the small improvement in median OS among patients with AML-MRC preselected to receive IC who received azacitidine, the overall response rate with azacitidine in the IC-preselection subgroup was only 27%, *vs*. 50% for patients who received IC. Response rates were slightly higher with azacitidine than with CCR in both cytogenetic risk groups, and response rates were higher within each treatment arm for patients with intermediate-risk cytogenetics compared with poor-risk cytogenetics (Table 3). Among patients aged ≥75 years, overall response rate was greater in the azacitidine arm (22.1% vs 10.1% with CCR).

**Table 3:** Response in patients with AML-MRC treated with azacitidine or CCR

All patients with AML-MRC (*N* = 262**)**				
	AZA (*n* = 129)		CCR (*n* = 133)	
	*n* (%)			
Overall Response Rate (CR + CRi)	32 (24.8)		23 (17.3)	
CR	25 (19.4)		20 (15.0)	
CRi	7 (5.4)		3 (2.3)	
Partial remission	1 (0.8)		2 (1.5)	
Stable disease	46 (35.7)		38 (28.6)	
Progressive disease	11 (8.5)		16 (12.0)	
	AML-MRC patients with Intermediate-risk cytogenetics		AML-MRC patients with Poor-risk cytogenetics	
	AZA (*n* = 63)	CCR (*n* = 61)	AZA (*n* = 66)	CCR (*n* = 72)
	n (%)			
Overall Response Rate (CR + CRi)	21 (33.3)	15 (24.6)	11 (16.7)	8 (11.1)
CR	18 (28.6)	13 (21.3)	7 (10.6)	7 (9.7)
CRi	3 (4.8)	2 (3.3)	4 (6.1)	1 (1.4)
Partial remission	1 (1.6)	1 (1.6)	0	1 (1.4)
Stable disease	25 (39.7)	18 (29.5)	21 (31.8)	20 (27.8)
Progressive disease	6 (9.5)	5 (8.2)	5 (7.6)	11 (15.3)
	AML-MRC patients ages 65–74 years		AML-MRC patients ages ≥ 75 years	
	AZA (*n* = 52)	CCR (*n* = 64)	AZA (*n* = 77)	CCR (*n* = 69)
	n (%)			
Overall Response Rate (CR + CRi)	15 (28.8)	16 (25.0)	17 (22.1)	7 (10.1)
CR	11 (21.2)	14 (21.9)	14 (18.2)	6 (8.7)
CRi	4 (7.7)	2 (3.1)	3 (3.9)	1 (1.4)
Partial remission	0	2 (3.1)	1 (1.3)	0
Stable disease	20 (38.5)	24 (37.5)	26 (33.8)	14 (20.3)
Progressive disease	5 (9.6)	6 (9.4)	6 (7.8)	10 (14.5)

AML-MRC, AML with myelodysplasia-related changes; AZA, azacitidine; CCR, conventional care regimens; CR, complete remission; CRi, CR with incomplete blood count recovery

Patients preselected to receive LDAC who were treated with azacitidine were more likely to have had a CR or CRi (27.2% *vs*. 13.9% with LDAC) and LDAC-treated patients were more likely to have progressive disease (13.9% *vs*. 4.9% with azacitidine) (Table 4). Interestingly, no patient with poor-risk cytogenetics in the azacitidine arm had progressive disease as their best response, compared with 9 of 46 patients (19.6%) treated with LDAC.

**Table 4:** Response among patients with AML-MRC preselected to receive LDAC and treated with azacitidine or LDAC

Patients with AML-MRC preselected to receive LDAC (n = 160)				
	AZA (*n* = 81)		LDAC (*n* = 79)	
	*n* (%)			
Overall Response Rate (CR + CRi)	22 (27.2)		11 (13.9)	
CR	17 (21.0)		10 (12.7)	
CRi	5 (6.2)		1 (1.3)	
Partial remission	1 (1.2)		1 (1.3)	
Stable disease	30 (37.0)		30 (38.0)	
Progressive disease	4 (4.9)		11 (13.9)	
	LDAC-preselected Intermediate-risk cytogenetics		LDAC-preselected Poor-risk cytogenetics	
	AZA (*n* = 47)	LDAC (*n* = 33)	AZA (*n* = 34)	LDAC (*n* = 46)
	*n* (%)			
Overall Response Rate (CR + CRi)	15 (31.9)	6 (18.2)	7 (20.6)	5 (10.9)
CR	12 (25.5)	6 (18.2)	5 (14.7)	4 (8.7)
CRi	3 (6.4)	0	2 (5.9)	1 (2.2)
Partial remission	1 (2.1)	1 (3)	0	0
Stable disease	19 (40.4)	15 (45.5)	11 (32.4)	15 (32.6)
Progressive disease	4 (8.5)	2 (6.1)	0	9 (19.6)
	LDAC-preselected age 65–74 years		LDAC-preselected age ≥ 75 years	
	AZA (*n* = 26)	LDAC (*n* = 38)	AZA (*n* = 55)	LDAC (*n* = 41)
	*n* (%)			
Overall Response Rate (CR + CRi)	10 (38.5)	7 (18.4)	12 (21.8)	4 (9.8)
CR	7 (26.9)	6 (15.8)	10 (18.2)	4 (9.8)
CRi	3 (11.5)	1 (2.6)	2 (3.6)	0
Partial remission	0	1 (2.6)	1 (1.8)	0
Stable disease	9 (34.6)	18 (47.4)	21 (38.2)	12 (29.3)
Progressive disease	2 (7.7)	4 (10.5)	2 (3.6)	7 (17.1)

*AML-MRC*, AML with myelodysplasia-related changes; *AZA*, azacitidine; *CR*, complete remission; *CRi*, CR with incomplete blood count recovery; *LDAC*, low-dose cytarabine

### Safety

The safety-evaluable cohort comprised 258 patients (99%). Grade 3–4 TEAEs were reported for 87% of patients in each treatment arm, and incidences of individual TEAEs were generally comparable between the azacitidine and CCR arms (Table 5). Rates of grade 3–4 haematological TEAEs decreased over time during continued treatment in both treatment arms (Table 6). TEAEs in patients with AML-MRC were generally consistent with those reported for all patients in AZA-AML [10].

**Table 5:** Most frequent (≥5% of patients) grade 3–4 treatment-emergent adverse events (TEAEs)

Preferred term	Azacitidine (*n* = 128) *n* (%)	CCR (*n* = 130) *n* (%)
Any grade 3–4 TEAE	111 (87)	113 (87)
Thrombocytopenia	33 (26)	27 (21)
Febrile neutropenia	29 (23)	43 (33)
Neutropenia	28 (22)	25 (19)
AML*	25 (20)	23 (18)
Pneumonia	24 (19)	18 (14)
Anaemia	19 (15)	21 (16)
Pyrexia	13 (10)	9 (7)
Hypokalaemia	9 (7)	10 (8)
Leukopenia	8 (6)	10 (8)
Sepsis	7 (6)	9 (7)
Decreased appetite	6 (5)	2 (2)
Dyspnoea	6 (5)	4 (3)

Safety-evaluable patients received at least 1 dose of study drug and had at least 1 post-baseline safety assessment. Patients who received BSC only were included in safety assessments if they had at least 1 post-randomisation safety assessment

*Worsening disease

**Table 6:** Occurrence of haematological Grade 3–4 adverse events across treatment cycles

Preferred term	Cycles 1–2		Cycles 3–4		Cycles 5–6		Cycles 7+	
	AZA (*N* = 128)	CCR (*N* = 130)	AZA (*n* = 89)	CCR (*n* = 61)	AZA (*n* = 75)	CCR (*n* = 25)	AZA (*n* = 57)	CCR (*n* = 17)
Any Grade 3–4 TEAE, *n* (%)	83 (65)	99 (76)	47 (53)	35 (57)	33 (44)	8 (32)	37 (65)	13 (77)
Febrile Neutropenia, *n* (%)	23 (18)	34 (26)	7 (8)	7 (12)	4 (5)	3 (12)	3 (5)	2 (12)
Thrombocytopenia, *n* (%)	24 (19)	26 (20)	11 (12)	8 (13)	8 (11)	0	9 (16)	0
Neutropenia, *n* (%)	16 (13)	20 (15)	10 (11)	9 (15)	3 (4)	2 (8)	12 (21)	5 (29)
Anaemia, *n* (%)	17 (13)	21 (16)	3 (3)	3 (5)	1 (1)	0	5 (9)	0

Safety evaluable patients received at least 1 dose of study drug and had at least 1 post-baseline safety assessment. Patients who received BSC only were included in safety assessments if they had at least 1 post-randomisation safety assessment

## Discussion

While slightly less than one-third of patients in the AZA-AML study were identified as having AML-MRC at local diagnosis, more than one-half of all patients were so identified upon central review. The discrepancy between local and central adjudication suggests that diagnosing dysplasia in routine clinical practice is a challenge, although dysplasia assessments are known to be subject to high interobserver variability [12]. As noted, 89 patients in AZA-AML with multilineage dysplasia determined in independent central review of BM aspirates were originally reported to have single-lineage or no dysplasia in local assessments, and 66 of these patients were not considered to have had AML-MRC at study entry. It is also noteworthy that only ~30% of centrally-defined AML-MRC cases were associated with locally reported prior MDS; a potential limitation of the current analysis was the inability to independently verify local reports of prior MDS. Potential under-recognition of prior MDS is consistent with results of a recent study indicating that the leukaemia cells of some older patients with apparent de novo AML harboured MDS-associated mutations, suggesting that such leukaemias may have had an unrecognised MDS prodrome [13].

The prognostic significance of morphologic dysplasia in AML has been analysed extensively. A preponderance of evidence suggests that dysplasia alone has no overall prognostic value [3, 14, 15], although within the AML-MRC classification, the presence of >50% micromegakaryocytes or >50% hypogranulated myeloid cells has been associated with poorer event-free survival [16]. Prognosis in AML-MRC may be better defined by the associated mutational profile; for example, in the presence of wild-type rather than mutated *NPM1* [17], or the presence of mutated *ASXL1* or *TP53* [18]. In the current analysis, patients with multilineage dysplasia only (i.e., no prior MDS or MDS-related cytogenetics) treated with azacitidine had much improved OS (16.3 months) compared with all AML-MRC azacitidine-treated patients (8.9 months), and compared with similar CCR-treated patients (7.1 months), suggesting that the presence of multilineage dysplasia alone may have prognostic value. Accurate morphologic assessment of multilineage dysplasia may be important for identifying the patients who might benefit most from azacitidine.

Even in this study of older patients with AML, the AML-MRC subgroup presented substantial treatment challenges; 57% of these patients were aged ≥75 years, 53% had poor-risk cytogenetics, and approximately one-third were reported to have had antecedent MDS. In the overall AZA-AML study population, only ~35% of patients had poor-risk cytogenetics, and as expected, proportionately fewer patients had antecedent MDS/MPN (~20%) [10]. Consistent with this prognostic profile, the median OS for AZA-AML patients treated with azacitidine was 8.9 months for patients with AML-MRC, compared with 10.4 months for all azacitidine-treated patients in the study. Likewise, median OS with CCR was 4.9 months for patients with AML-MRC compared with 6.5 months among all CCR-treated patients. Nevertheless, the relative survival benefit for azacitidine observed overall in the study was also maintained in the AML-MRC population, with a significant improvement in median OS of 4.0 months and a 26% reduced risk of death compared with CCR. Additionally, median OS with azacitidine was almost double that of CCR in patients with intermediate-risk cytogenetics and among “younger” patients (ages 65–74 years) compared with CCR.

This analysis also suggested an OS benefit with azacitidine in the subgroup of patients preselected to receive low-intensity LDAC treatment before randomisation. Median OS with azacitidine was more than twice that with LDAC in patients with AML-MRC. Keeping in mind that comparisons between azacitidine and LDAC by age and cytogenetic risk comprised relatively small patient subgroups, median OS was more than twice as long for LDAC-preselected patients with intermediate-risk cytogenetics treated with azacitidine, and almost 3-fold longer for patients aged 65–74 years. While they did not fare as well, azacitidine-treated patients with poor-risk cytogenetics, and those aged ≥75 years, had 2- to 5-month improvement in median OS compared with LDAC-treated patients. LDAC has previously been shown to be ineffective in patients with AML with adverse cytogenetics [19]. It should be noted that better outcomes for azacitidine-treated patients in this preselection group may in part reflect differences in treatment exposure; the median number of azacitidine treatment cycles for this population was 3 times that of LDAC treatment cycles (6 vs 2 cycles). Nevertheless, compared with all LDAC-treated patients in AZA-AML (*n* = 158), LDAC-treated patients with AML-MRC (*n* = 79, 50%) had a somewhat lower median OS (6.4 vs 4.6 months, respectively), suggesting that LDAC may not be optimal in this patient population. Another approach that has shown promise in a partially overlapping patient population to that evaluated in this study is CPX-351 (a liposomal formulation of cytarabine and daunorubicin at a fixed 5:1 M ratio) which has been compared in a phase 3 study to standard “7 + 3” induction chemotherapy in 309 patients aged 60–75 years with previously untreated sAML, tAML, or AML with MDS-related cytogenetic abnormalities [20]. CPX-351 treatment resulted in a median OS of 9.6 months compared with 6 months with standard induction chemotherapy (*P* = 0.005) in this somewhat younger cohort who were considered fit for standard induction therapy.

Overall response rates in azacitidine-treated patients ranged from 17 to 33% in these analyses, and were higher than response rates with CCR. Interestingly, the difference between azacitidine and CCR overall response rates in AML-MRC (7.5% improvement with azacitidine) was larger than was the difference seen for all patients in the AZA-AML study (2.7% in favour of azacitidine) [10]. Similarly, the significant 13.3% improvement in overall response rate with azacitidine compared with LDAC in LDAC-preselected patients with AML-MRC was much larger than was the difference in response rates for all AML patients in the LDAC preselection group in AZA-AML (1.4% improvement with azacitidine vs LDAC) [21]. Overall response rate with azacitidine in the current AML-MRC analyses was similar to that for all azacitidine-treated patients in AZA-AML (27.8%) [10]; in contrast, the overall response for LDAC-treated patients with AML-MRC (13.9%) is lower than that reported for all LDAC-treated patients in AZA-AML (25.9%) [21]. This suggests that LDAC may be less effective in AML-MRC than in other types of AML.

The safety profile of azacitidine in patients with AML-MRC was similar to that of all azacitidine-treated patients in AZA-AML [10].

## Conclusions

Safety and efficacy results of the current analyses indicate that patients with AML-MRC may fare better if treated with azacitidine rather than with CCR, and more specifically, rather than with LDAC. These data also suggest that diagnostic improvement is needed to more accurately identify patients with preleukaemic haematological disorders, and to recognise multilineage dysplasia. Once AML-MRC is diagnosed, azacitidine may be the treatment of choice for patients who are not candidates for induction chemotherapy or stem cell transplantation.

## Abbreviations

95%CI: 95% confidence interval
AML: Acute myeloid leukaemia
AML-MRC: AML with myelodysplasia-related changes
AML-NOS: AML not otherwise specified
AZA-AML: AZA-AML-001 phase 3 trial
BM: Bone marrow
BSC: Best supportive care
CCR: Conventional care regimens
CR: Complete remission
CRi: CR with incomplete haematologic recovery
ECOG PS: Eastern Cooperative Oncology Group performance status
HR: Hazard ratio
IC: Intensive chemotherapy
IWG: International Working Group
LDAC: Low-dose cytarabine
MDS: Myelodysplastic syndromes
MedDRA: Medical Dictionary for Regulatory Activities
NCCN: National Comprehensive Cancer Network
OS: Overall survival
sAML: Secondary AML
tAML: Therapy-related AML
TEAE: Treatment-emergent adverse event
WBC: White blood cell
WHO: World Health Organization
